# Parenthood and factors that influence outdoor recreational physical activity from a gender perspective

**DOI:** 10.1186/1471-2458-11-93

**Published:** 2011-02-10

**Authors:** Katarina Sjögren, Eva Ekvall Hansson, Louise Stjernberg

**Affiliations:** 1 Faculty of Medicine, Department of Clinical Sciences in Malmö, General Practice, Lund University, CRC entrance 72:28:11, Malmö University Hospital, 205 02 Malmö, Sweden; 2 School of Health Science, Blekinge Institute of Technology, 371 79 Karlskrona, Sweden

## Abstract

**Background:**

A physically active life promotes both physical and mental health, increasing well-being and quality of life. Physical activity (PA) performed outdoors has been found to be particularly good for promoting well-being. However, participation in PA can change during the course of a lifetime. Parenthood has been found to be a life event associated with decreased PA, especially among women, although studies in the field are sparse. The aim of this study was to investigate participation in outdoor recreational PA, and factors influencing participation among parents-to-be, with and without previous children, from a gender perspective.

**Methods:**

This study included baseline data from parents-to-be, 224 women and 208 men, from the municipality of Karlskrona in south-east Sweden. Data collection was carried out during 2008-2009. We measured the self-reported amount of outdoor recreational PA undertaken during the last year and analysed the probability of participating in this PA using 25 variables covering individual and socioeconomic factors.

**Results:**

Seventy-six per cent of the women and 65% of the men had participated in outdoor recreational PA, varying from several times per month to every day, over a 12-month period prior to one month before pregnancy. Participation in PA indoors and owning a dog or a horse emerged as the most important factors associated with the probability of participation in outdoor recreational PA. Men were affected by a greater number of factors than women, for example men who had a family situation that permitted outdoor recreational PA participated in activities to a greater extent than men without such a family situation. The physical aspect, i.e. improved physical condition, staying power and vigour, also played a significant role with regard to participation among men.

**Conclusions:**

Becoming a parent is a life-changing event that affects participation in PA. By offering family-oriented PA choices that involve both parents and children, midwives and health promoters can encourage parents to be active and to support each other. The promotion of outdoor recreational PA, which also has restorative effects on well-being, needs to focus on activities which are attractive and affordable for the majority of both women and men.

## Background

Being physically active promotes both physical and mental health, increasing well-being and quality of life [1,2]. Physical activity (PA), defined as "any bodily movement produced by the contraction of skeletal muscle that increases energy expenditure above a basal level" [3], is associated with a wide range of health benefits, including mitigation of chronic health problems as well as improvements in mental health and well-being [2]. Participation in PA outdoors promotes well-being and evidence has been found for a positive relationship between green space and self-perceived health [4-6]. It has also been found to have restorative effects on human health and a reduction in mental fatigue [7,8]. Outdoor recreational PA is defined here as being outside in natural or cultural landscapes for the purpose of well-being and encounters with nature without any demand for competition [9].

Despite the well-documented health benefits of PA, inactivity was identified in 2009 as the fourth leading cause of chronic diseases and mortality globally and contributes to more than three million preventable deaths worldwide [10]. According to Sjöström et al [11] only 23% of the Swedish adult population are sufficiently active for optimal health benefits. The situation is similar in other western European countries and the United States. In general, women tend to be less physically active than men [1,11,12].

Participation in PA can change over a lifetime and from a public health perspective it is important to understand how life events may impact on participation in PA. Periods of life transition may be associated with changes in health-related behaviour such as PA [13]. Parenthood is a life event associated with decreased PA, especially among women [14,15]. A review of PA and parenthood by Bellows-Riecken and Rhodes [15] shows a negative relationship between parenthood and participation in PA in 14 of the 17 studies examined. Brown et al [16] found women with children performed less PA than women without children and Schmitz et al [17] found that parenthood resulted in reduced PA in women but not in men, with the biggest difference occurring during first parenthood. Lack of time and social support, fatigue, childcare, and other obligations have been identified as factors that impacted negatively on parents' participation in PA [18-21].

As parents play an important role in the socialisation and development of PA patterns in their children, knowledge of determinants that affect participation in PA among both parents as well as parents-to-be is necessary to develop effective health-promotion programmes. Studies in the field are sparse, and there is a particular lack of data about differences in PA patterns between men with and without children, as well as between women and men with children [15]. The aim of this study therefore was to investigate participation in outdoor recreational PA, and factors influencing participation among parents-to-be, with and without previous children, from a gender perspective.

## Methods

### Study area, participants, and design

This study includes baseline data from parents-to-be with and without previous children. Those who already had children are in this study named either "parents" or "women/men with children" and those who did not have previous children and were now expecting their first child are either named "non-parents" or "women/men without children". The data were collected between March 2008 and February 2009 from the municipality of Karlskrona. Karlskrona, an urban region with 62,900 inhabitants, is located in south-east Sweden on the Baltic Sea and both woodland and archipelago settings are readily accessible.

On finding themselves pregnant, Swedish women book an initial meeting with a midwife through an antenatal clinic, in most cases at 8-10 weeks of pregnancy. Parents-to-be who contacted either of the two antenatal clinics in Karlskrona were invited by the midwife to participate in this study. If they agreed to participate in the study, separate questionnaires (with different colours) were sent by post to the woman and her partner, and were returned to the midwife at the initial meeting.

The majority of the questions had been applied and validated in the Swedish Survey of Living Conditions (ULF) and in a national survey about outdoor life and nature tourism in Sweden [9]. Some extra added questions were tested in a pilot study (n = 10) and there was no need for adjustment before use. Identical questions were used for both women and men, and the respondents were required to address their situation one month before pregnancy. The questionnaires, which were filled in retrospectively, investigated age, cohabitation, level of education, personal financial situation, living conditions, dog or horse ownership, tobacco and alcohol consumption, and previous children and their age. Questions about outdoor recreational PA concerned activities during the previous 12 months (calculated from one month before pregnancy) and reasons for participation (i.e. to benefit for physical and/or social reasons and/or the adventure and/or restorative effects and also to attain well-being).

The questionnaires also included questions about obstacles to participating in outdoor recreational PA (abandoning outdoor activities during the evenings due to fear of violence, cost, the experience of activities being too demanding physically, limitations due to the family situation, illness/functional disorder) and other considerations (lack of appropriate areas to perform it in, equipment, time, courage, interest, or too little knowledge about how to perform activities). The participants also answered questions about indoor PA during the previous 12 months. The inclusion criteria were pregnant woman who spoke Swedish. The exclusion criteria were woman who had experienced miscarriage or other complications during previous pregnancies or deliveries or if the midwife did not consider it suitable to ask about participation in the study due to the woman's general state of health.

Outdoor recreational PA was measured by the survey question "How often have you participated in outdoor recreational PA during the last 12 months?" Participants were asked to mark activities participated in during the last 12 months from a list of 24 outdoor recreational activities from a Swedish national survey dealing with outdoor recreational life [9]. This list included a wide spectrum of physical and more sedentary activities, as we wished to include activities with a restorative effect on well-being as well as activities associated with physical effort. The following activities were presented: strolling in the woods and fields, walking, jogging, orienteering, Nordic walking, golf, biking, rollerblading, bathing, diving, canoeing, sailing, angling, motorboating, skating, cross-country skiing, downhill skiing, tobogganing, sunbathing, hunting, cross-country riding, bird-watching, camping in the countryside and gardening.

### Statistical analysis

In the statistical analyses a chi-squared test was used to compare proportions between groups. When the expected frequencies were < 5 in any group, Fisher's exact test for the comparison of two proportions was used. Multivariate analyses were carried out by means of logistical regression models (method: backward conditional), with participation in outdoor recreational PA as the dependent variable. Participation in outdoor recreational PA was dichotomised into "active" (if a person participated in activities several times (> 3 times per month) a month to every day) and "not active" (if a person never participated in activities or up to 2-3 times per month).

Simultaneous relationships between the dependent variable and all independent variables were modelled and evaluated using odds ratios (ORs). Two different models were tested separately for 1) women with children, 2) women without children, 3) all women, 4) men with children, 5) men without children, and 6) all men. In the first model, all independent variables from the questionnaire were included. The second model included variables from the first model for which a significant impact on the probability of participation in outdoor recreational PA existed between the independent variables that included more than five participants in each combined group. The second model also evaluated the interactions between these variables, with a p-value < 0.005. The statistical significance of the variables was determined using a 95% confidence interval (CI). Data were computerised and analysed using SPSS 17.0 for Windows. This study was approved by the Regional Ethical Review Board in Lund, Sweden.

## Results

During the study period, 669 women were registered at the two local antenatal clinics. Of these, 431 met the inclusion criteria and upon inclusion the women ranged from week six to week 33 of pregnancy (mean 11 weeks). The mean age of non-participating women was 30 years. The reasons for non-participation were unwillingness, unsuitability for study inclusion (as determined by the midwife), and non-compliance with the inclusion criteria. A total of 224 women and 208 men agreed to participate. Of the women, 121 had children, and of those, 115/121 (95%) had children aged 0-5 years. Of the men, 113 had children, and of those, 106/113 (94%) had children aged 0-5 years. Of the women included, 171/224 (76%) had participated in outdoor recreational PA several times per month to every day during the last 12 months up to one month before pregnancy, and 135/208 (65%) men had participated in activities several times per month to every day during the same period. Outdoor recreational activities had a positive impact on feelings of well-being in 221/224 (99%) women and 198/208 (95%) men. More than half of the women and men had a higher education and the majority of them were able to cover an unexpected cost within a week (Table 1). The demographic characteristics of study participants are presented in Table 1.

**Table 1:** Participant characteristics one month before pregnancy

	Men with children	Men without children	All men	Women with children	Women without children	All women
Variables (%)	n = 113 (%)	n = 95 (%)	n = 208 (%)	n = 121 (%)	n = 103 (%)	n = 224 (%)
Mean age	34	31	32	31	28	30
Cohabiting^1^	102 (99)	94 (99)	206 (99)	119 (98)	102 (99)	221 (99)
Lower education^2^	49 (43)	40 (42)	89 (43)	55 (45)	42 (41)	97 (43)
Higher education^3^	64 (57)	55 (58)	119 (57)	66 (55)	61 (59)	127 (57)
Personal financial situation^4^	100 (89)	83 (87)	183 (88)	102 (84)	88 (85)	190 (85)
Living in the countryside^5^	49 (43)	24 (25)	73 (35)	52 (43)	27 (26)	79 (35)
Living in a town^6^	64 (57)	71 (75)	135 (65)	69 (57)	76 (74)	145 (65)
Owning a dog or a horse	31 (27)	17 (18)	48 (23)	32 (26)	20 (19)	52 (23)
Being a smoker^7^	22 (20)	18 (19)	40 (19)	16 (13)	27 (26)	43 (19)
Using snuff^8^	43 (38)	34 (36)	77 (37)	2 (2)	3 (3)	5 (2)
Drinking alcohol^9^	80 (70)	79 (83)	159 (77)	65 (51)	66 (64)	128 (57)
Drinking no alcohol	33 (30)	16 (17)	49 (23)	59 (49)	37 (36)	96 (43)

^1^married/living with a partner

^2^completion of nine-year compulsory school, senior high-school

^3^completion of university education

^4^able to cover an unexpected cost of 14 000 Swedish crowns (approximately 1400 Euros) within one week

^5^a small village in the countryside with mainly single-family houses

^6^a densely built-up area, in a quarter of a town or in a suburb

^7^smokes regularly/sometimes

^8^using snuff (a Swedish smokeless tobacco) regularly/sometimes

^9^drinking 1-9 glasses/week

All variables from the questionnaire are shown in Table 2, and Table 3 contains participation in different kinds of PA during the last 12 months. All those who participated in non-physical strenuous activities also participated in at least one physical activity. Women strolled and walked significantly more (Pearson chi-square = 5.724, *p *= 0.017) than men irrespective of whether or not they had children. In all other activities studied, no significant differences were seen between women and men. Women with children participated in significantly more winter sports (Pearson chi-square = 6.213, *p *= 0.013) and gardening (Pearson chi-square = 9.417, *p *= 0.009) than women without children, and women without children participated in significantly more PA indoors (Pearson chi-square = 14.877, *p *< 0.001) than women with children. Men with children participated in significantly more gardening (Pearson chi-square = 20.799, *p *< 0.001) than men without children, and men without children participated in significantly more PA indoors (Pearson chi-square = 7.687, *p *= 0.006).

**Table 2:** All independent variables included in the first model.

**Variables**	**Coded as**	
	**0**	**1**
	
Cohabiting	yes^1^	no^2^
Level of education	lower^3^	higher^4^
Personal financial situation	able^5^	not able^6^
Living conditions	countryside^7^	town^8^
Owning a dog or horse	yes	no
Smoking habits	smoker^9^	non-smoker^10^
Snuff habits	snuffer^11^	non-snuffer^12^
Alcohol habits	drinker^13^	non-drinker^14^
Able to participate in as much outdoor recreational PA as wanted	yes	no
Participated in outdoor recreational PA for physical reasons	yes	no
Participated in outdoor recreational PA for social reasons	yes	no
Participated in outdoor recreational PA for the adventure	yes	no
Participated in outdoor recreational PA for well-being	yes	no
Not able to participate in as much outdoor recreational PA as wanted due to costs	yes	no
Not able to participate in as much outdoor recreational PA as wanted due to physical level	yes	no
Not able to participate in as much outdoor recreational PA as wanted due to family situation	yes	no
Not able to participate in as much outdoor recreational PA as wanted due to illness/functional disorder	yes	no
Not able to participate in as much outdoor recreational PA as wanted due to lack of appropriate areas	yes	no
Not able to participate in as much outdoor recreational PA as wanted due to lack of knowledge	yes	no
Not able to participate in as much outdoor recreational PA as wanted due to lack of equipment	yes	no
Not able to participate in as much outdoor recreational PA as wanted due to lack of time	yes	no
Not able to participate in as much outdoor recreational PA as wanted due to lack of courage	yes	no
Not able to participate in as much outdoor recreational PA as wanted due to lack of interest	yes	no
Participated in PA indoors	yes	no
Abandoned outdoor recreational PA during the evening due to fear of violence	yes^15^	no^16^

The logistic regression analysis evaluated factors with a significant influence on the participation in outdoor recreational physical activity (PA).

^1^married/living with a partner

^2^single or living apart

^3^completed nine-year compulsory school, senior high school

^4^completed university education

^5^able to cover an unexpected cost of 14 000 Swedish crowns (approximately 1400 Euros) within one week

^6^not able to cover an unexpected cost of 14 000 Swedish crowns within one week

^7^living in a small village in the countryside with mainly single-family houses

^8^living in a densely built-up area, in a quarter of a town, or in a suburb

^9^smokes regularly/sometimes

^10^stopped smoking/never smoked

^11^using snuff (smokeless tobacco) regularly/sometimes

^12^stopped using snuff/never used it

^13^drinking 1-9 glasses/week

^14^drinking no alcohol

^15^often/rather often

^16^never/seldom

**Table 3:** Outdoor recreational physical activities carried out during the last 12 months

	Men with children	Men without children	All men	Women with children	Women without children	All women
Factor	n = 113 (%)	n = 95 (%)	n = 208 (%)	n = 121 (%)	n = 103 (%)	n = 224 (%)
Outdoor recreational PA*^1^	71 (63)	64 (67)	135 (65)	87 (72)	84 (82)	171 (76)
Strolling/walking^2^	46 (41)	33 (35)	79 (38)	58 (52)	57 (55)	115 (51)
Exercising^3^	42 (37)	46 (48)	88 (42)	53 (44)	51 (50)	104 (46)
Aquatic sports^4^	46 (41)	48 (51)	94 (45)	62 (51)	51 (50)	113 (50)
Winter sports^5^	17 (15)	11 (12)	28 (14)	21 (17)	6 (6)	27 (12)
Gardening	79 (70)	37 (39)	116 (56)	70 (58)	39 (38)	109 (49)
Indoor PA*^6^	44 (39)	63 (66)	107 (51)	39 (32)	61 (59)	100 (45)
Participation in non-strenous activities^7^	55 (49)	60 (63)	115 (55)	78 (65)	58 (56)	136 (61)

^1^participated in several times per month to every day during the last 12 months, all activities below included

^2^strolling, walking, Nordic walking, golf

^3^jogging, rollerblading, biking, orienteering, cross-country riding

^4^canoeing, diving, bathing

^5^cross country skiing, skating, downhill skiing, tobogganing

^6^floorball, weight training, aerobics, swimming, gymnastics

^7^hunting, sunbathing, angling, camping, bird watching, motorboating, sailing

*Physical activity

### Differences between groups and factors significantly associated with participation in outdoor recreational PA among women

Factors significantly associated with outdoor recreational PA among all women with and without children were ownership of a dog or horse (Fisher's exact test *p *< 0.001), participating because of well-being (Fisher's exact test *p *= 0.022), and participating in activities indoors (Pearson chi-square = 11.366, *p *= 0.001). The only factor that significantly enhanced participation in outdoor recreational PA among women with children was ownership of a dog or horse (Fisher's exact test *p *< 0.001). Among women with no children, a higher level of education (Pearson chi-square = 4.833, *p *= 0.028), dog or horse ownership (Fisher's exact test *p *= 0.018), participation in outdoor recreational PA because of the feeling of well-being (Fisher's exact test *p *= 0.003), and participation in PA indoors (Pearson chi-square = 10.447, *p *= 0.001) were factors that significantly enhanced participation in outdoor recreational PA.

### Differences between groups and factors significantly associated with participation in outdoor recreational PA among men

Factors that were associated significantly with outdoor recreational PA among all men, with and without children, were a higher level of education (Pearson chi-square = 8.220, *p *= 0.004), dog or horse ownership (Pearson chi-square = 11.527, *p *= 0.001), participation in PA for physical reasons (Pearson chi-square = 19.859, *p *< 0.001), participation for the adventure (Pearson chi-square = 9.888, *p *= 0.002), access to appropriate areas to perform outdoor recreational PA (Fisher's exact test *p *= 0.026), interest (Pearson chi-square = 4.928, *p *= 0.026), and participation in PA indoors (Pearson chi-square = 20.439, *p *< 0.001).

Factors that significantly enhanced participation in outdoor recreational PA among men with children were dog or horse ownership (Pearson chi-square = 5.804, *p *= 0.016), participation in PA for physical reasons (Pearson chi-square = 10.880, *p *= 0.001), participation for the adventure (Pearson chi-square = 10.108, *p *= 0.001), and participation in PA indoors (Pearson chi-square = 6.435 *p *= 0.011). Factors that significantly enhanced participation in outdoor recreational PA among men with no children were a higher education level (Pearson chi-square = 9.481, *p *= 0.002), living in a town (Pearson chi-square = 4.407, *p *= 0.036), dog or horse ownership (Fisher's exact test *p *= 0.009), participation in PA for physical reasons (Pearson chi-square = 8.548, *p *= 0.003), time availability (Pearson chi-square = 4.214, *p *= 0,040), interest (Pearson chi-square = 5.766, *p *= 0.016), and participation in PA indoors (Pearson chi-square = 15.699, *p *< 0.001).

### Factors predicting the probability of participation in outdoor recreational PA among men and women

Variables significantly associated with an increased probability of participating in outdoor recreational PA among all women and men, irrespective of whether or not they had children, are presented in Table 4. No factors were significantly associated with the probability of participating in outdoor recreational PA among women with or without children in the final models. Our data indicate that for women, irrespective of whether they had children or not, dog or horse ownership, the interaction of dog or horse ownership and the ability to cover an unexpected cost, as well as participation in PA indoors, were factors significantly associated with an increased probability of participation in outdoor recreational PA.

**Table 4:** Binary logistic regression predicting independent factors significantly associated with the probability of participating in outdoor recreational physical activity

	Men with children			Men without children			All men			All women		
Factors	OR	CI (95%)	p	OR	CI (95%)	p	OR	CI (95%)	p	OR	CI (95%)	p
Higher education level	n.s.	n.s.	n.s.	4.211	1.316-13.473	0.015	n.s.	n.s.	n.s.	n.s.	n.s.	n.s.
Dog or horse ownership	n.s.	n.s.	n.s.	n.s.	n.s.	n.s.	3.419	1.167-10.019	0.025	24.523	3.220-186.778	0.002
Permissive family situation*	n.s.	n.s.	n.s.	n.s.	n.s.	n.s.	3.084	1.206-7.884	0.019	n.s.	n.s.	n.s.
Dog or horse ownership and physical reasons†	5.214	1.123-6.897	0.001	13.583	2.964-62.240	0.001	5.570	2.405-12.904	0.001	n.s.	n.s.	n.s.
Dog or horse ownership and indoor activities‡	n.s.	n.s.	n.s.	13.585	3.327-55.476	0.001	n.s.	n.s.	n.s.	n.s.	n.s.	n.s.
Dog or horse ownership and economy#	n.s.	n.s.	n.s.	n.s.	n.s.	n.s.	n.s.	n.s.	n.s.	2.705	1.021-7.168	0.045
Indoor activities¶	2.783	1.430-9.967	0.027	n.s.	n.s.	n.s.	n.s.	n.s.	n.s.	4.047	1.929-8.488	0.001

*Having a family situation that allowed participation in outdoor recreational physical activity

†Interaction variable measuring dog or horse ownership and participation in outdoor recreational physical activity for physical reasons

‡ Interaction variable measuring dog or horse ownership and participation in physical activity indoors

# Interaction variable measuring dog or horse ownership and ability to cover an unexpected cost

¶Participation in physical activity indoors

OR, odds ratio; CI, confidence interval; n.s., not significant

For men, irrespective of child status, dog or horse ownership, the interaction of dog or horse ownership and participation in PA for physical reasons and a family situation that permitted outdoor recreational PA were factors significantly associated with an increased probability of participating in outdoor recreational PA. For men without children, the interaction between dog or horse ownership and participation in PA indoors, the interaction between dog or horse ownership and participation in PA for physical reasons, and higher levels of education were factors significantly associated with an increased probability of participation in outdoor recreational PA. For men with children, the interaction between dog or horse ownership and participating in PA for physical reasons, and participation in PA indoors significantly increased the probability of participating in outdoor recreational PA.

## Discussion

The main findings indicate that the majority of the women and men had participated in outdoor recreational PA several times a month to every day during the 12-months prior to one month before pregnancy. More factors came into effect if men were physically active outdoors compared to women. However, for both women and men, having a dog or a horse seemed to be the most important factor associated with the probability of participation in outdoor recreational PA. Participation in PA indoors was also associated with participation in outdoor recreational PA for both women and men. Economic situation was significant when analysing all women together. For men, irrespective of whether they had children or not, the physical aspect, i.e. improved physical condition, staying power and vigour, played a significant role. For all men, a family situation that allowed PA was important to participation in outdoor recreational PA.

PA is a wide-ranging term that includes a variety of activities that may occur in many different settings and modes and at varying frequencies and intensities. This complexity makes it difficult to measure. However, our definition of PA included a broad range of activities. Participants were given examples of activities in the questionnaire and were allowed to evaluate the various activities that they participated in over the course of a year. The intensity and type of activity can vary widely during a year; the seasons are likely to influence both the level of activity and the chosen activity [22].

Participants reported only how often they had participated in outdoor recreational PA during the previous 12 months (up to one month before pregnancy) and not the intensity or time, which would have provided more information about their habits. Bias may exist due to the retrospective nature of the study. Data collection at the population level often involves self-reported measures through the use of questionnaires. This study strategy is frequently employed for reasons of practicality, low cost, and general acceptance [23]. Self-reports are useful for gaining insight into PA patterns in populations although there is always a risk of over- and underestimation of the answers. Indeed, the measurement method may have a significant impact on the observed levels of PA; self-reported measures of PA were both higher and lower than those measured directly [24]. Even self-reports about smoking and alcohol consumption may cause systematic errors and should be treated with caution [25,26].

The participants in this study included the majority of pregnant women and their partners within the defined geographical area. However, since the rate of non-participation was fairly high (48%), generalising the findings must be done with caution. Non-response can affect the validity of epidemiological studies and introduce bias [27]. Response quality is also important in the sense that incomplete or incorrectly-answered questionnaires will have the same effects as a poor response. The questionnaires were completed very well by the participants in this study. Accordingly, the internal validity was very low, which must be regarded as a strength of the study. The participants probably remembered whether or not they participated in outdoor recreational PA during the last 12 months although the number of times might be misleading. Other strengths are that we included both women and men and that the distribution according to gender was almost equal, as well as the distribution of both women and men with and without children. The classification of the different outdoor recreational physical activities carried out during the last 12 months was at first made by all the authors separately and then compared. There was no disagreement among the authors about the classification.

In contrast to previous studies [1,11,12], which stated that women tend to be less physically active than men, 76% of the women and 65% of the men included in our study participated in outdoor recreational PA during the 12-month period prior to one month before pregnancy. This observation may be explained by our inclusion of a wider spectrum of outdoor activities, not only activities strongly associated with sports and exercise that are traditionally pursued mostly by men. Women, both with and without children, were found to stroll and walk significantly more than men. These findings are in agreement with previous work [28] demonstrating that women of all ages tend to walk more than men.

Dog or horse ownership emerged as a factor that was strongly associated with participation in outdoor recreational PA among both men and women in this study. We chose to focus on dogs and horses, since these animals require exercise and involve outdoor recreational PA for the owner. Our findings corroborate other studies [29], demonstrating that dog owners spend more time in mild to moderate PA compared to non-dog owners. We also found that men who had a dog or a horse and participated in outdoor recreational PA for physical reasons were more capable of being active. This finding might indicate that men who have an interest in PA for physical reasons tend to have a dog or a horse. Since having pets, especially a horse, involves additional expense, people in a better financial situation are more likely to have a dog or a horse. Along the same lines, we noted that women who were able to cover an unexpected cost and had a dog or a horse participated more in outdoor recreational PA than women who did not. However, some of the groups that this data refer to have less than five participants and caution may be needed when drawing conclusions.

Participation in PA indoors was associated with participation in outdoor recreational PA, both for women and men. A trend towards polarisation can be seen, in which the population is divided into those who perform PA and those who do not [30]. Accordingly, it seems that people active in PA are active independent of indoor or outdoor activities. Both women and men without children participated in PA indoors significantly more than those who had children. This occurrence may be explained by the fact that healthcare PA is easier to perform outdoors with children than indoors. This finding underscores the importance of midwives informing parents-to-be of the positive health effects of outdoor recreational PA, especially since the majority of women and men reported that outdoor recreational activities had a positive impact on their well-being. Other studies [4-6] have also shown a positive relationship between green space and self-perceived health. PA that is not physically strenuous e.g. sunbathing, bird watching and hunting (we have defined hunting based on Swedish conditions, where hunting mostly means sitting and waiting for the animal to show up), were among the most widely pursued activities among both women and men. These types of activity may have a restorative effect on people's health and should not be underrated [8].

In Sweden, as in many other countries, there are differences in participation in PA among different groups in society. Lower levels of PA are more common among individuals with lower education levels than among those with a higher level of education [31,32]. In this study, men without children and with a higher level of education were more likely to perform outdoor recreational PA than those with a lower level of education.

Men, irrespective of whether they had children or not, who had a family situation that permitted outdoor recreational PA, participated in activities to a greater extent than men without such a family situation. As previous work has mainly focused on the PA of women, to our knowledge no similar findings have been reported. Previously, women listed lack of time, social support, family commitments and role obligations as barriers to their participation in PA [18-21], factors which are included in the family situation. Our findings thus indicate a need for more research focusing on PA of men who become parents.

Contradictory reports exist regarding the impact of age and number of children on participation in PA [33-37]. We focused only on the differences between women and men with and without children; accordingly, we have not studied how age and number of children may affect participation in outdoor recreational PA.

Becoming a parent is a life-changing event that affects participation in PA, probably due to lifestyle changes. Pregnancy and parenthood have been found to be life events associated with decreased PA [14,15]. The decline is seen mostly among women, possibly explained by a research focus on women's perception of and influences on their children's PA patterns. Bellows-Riecken and Rhodes [15] reviewed 31 studies published from 1989 through to July 2007 that dealt with the impact of parenthood on PA. Of the 25 independent samples, 17 focused on women, seven included both women and men, and only one was based solely on men. This review, in addition to the results from this study, demonstrate the need for a greater focus on men's PA during this life transition, as both parents play an important role in shaping their children's routines (including PA habits). There would generally appear to be a stronger influence of fathers' PA patterns on both boys and girls [38].

## Conclusions

The majority of the women and men had participated in outdoor recreational PA from several times per month up to every day during the 12-month period prior to one month before pregnancy. More factors affected whether men were physically active than women. Dog or horse ownership seemed to be an important factor associated with the probability of participating in outdoor recreational PA. Those active in PA are also active independent of indoor or outdoor activities. Our findings also indicate that becoming a parent is a life-changing event that affects participation in PA. Midwives and health promoters have an important role to play, encouraging both parents to be active and to support each other by offering family-oriented PA choices that involve parents and children or that provide an opportunity for parents to perform PA while their children are involved in their own activities. Further studies dealing with gender differences are needed in this field. The promotion of outdoor recreational PA, which also has restorative effects on well-being, needs to focus on activities that are attractive and affordable for the majority of both men and women.

## Competing interests

The authors declare that they have no competing interests.

## Authors' contributions

KS and LS were responsible for the conception, design and acquisition of data. KS was responsible for the analysis and interpretation of data and drafting the initial manuscript. LS and EEH were responsible for reviewing all drafts of the manuscript. All authors read and approved the final manuscript.

## Pre-publication history

The pre-publication history for this paper can be accessed here:

http://www.biomedcentral.com/1471-2458/11/93/prepub
